# Identification and characterization of a cyclosporin binding cyclophilin from Staphylococcus aureus Newman

**DOI:** 10.6026/97320630013078

**Published:** 2017-03-31

**Authors:** Soumitra Polley, Soham Seal, Avisek Mahapa, Biswanath Jana, Anindya Biswas, Sukhendu Mandal, Debabrata Sinha, Keya Sau, Subrata Sau

**Affiliations:** 1 Department of Biochemistry, Bose Institute, Kolkata, West Bengal, India;; 2 Department of Biotechnology, Haldia Institute of Technology, Haldia, West Bengal, India;

**Keywords:** Staphylococcus aureus, cyclophilin, cyclosporin A, GdnCl, unfolding, intermediate, stability

## Abstract

Cyclophilins, a class of peptidyl-prolyl cis-trans isomerase (PPIase) enzymes, are inhibited by cyclosporin A (CsA), an immunosuppressive drug. Staphylococcus aureus Newman, a pathogenic bacterium, carries a gene for encoding a putative cyclophilin (SaCyp). SaCyp shows significant homology with other cyclophilins at the sequence level. A three-dimensional model structure of SaCyp harbors a binding site for CsA. To verify whether SaCyp possesses both the PPIase activity and the CsA binding ability, we have purified and investigated a recombinant SaCyp (rCyp) using various in vitro tools. Our RNase T1 refolding assay indicates that rCyp has a substantial extent of PPIase activity. rCyp that exists as a monomer in the aqueous solution is truly a cyclophilin as its catalytic activity specifically shows sensitivity to CsA. rCyp appears to bind CsA with a reasonably high affinity. Additional investigations reveal that binding of CsA to rCyp alters its structure and shape to some extent. Both rCyp and rCyp-CsA are unfolded via the formation of at least one intermediate in the presence of guanidine hydrochloride. Unfolding study also indicates that there is substantial extent of thermodynamic stabilization of rCyp in the presence of CsA as well. The data suggest that rCyp may be exploited to screen the new antimicrobial agents in the future.

## Background

A newly synthesized polypeptide becomes functional only when it is folded correctly. In the live cells, many chaperones and isomerases are usually involved in the folding of nascent polypeptides [1]. Enzymes like peptidyl-prolyl cis-trans isomerases (PPIases) stimulate the folding of polypeptides primarily by catalyzing the cis-trans isomerization of peptide bonds preceding the proline residues. Despite the well-defined catalytic activity, currently little is known about the exact cellular substrates of these enzymes. Several reports have, however, demonstrated that PPIases are involved in the variety of cellular functions such as signal transduction, transcriptional regulation, cell differentiation, protein secretion, and apoptosis [1]. In addition, the links of PPIases in many diseases have been clearly established.

PPIase enzymes carry either single domain or multiple domains and are located in the membrane, cytosol, and in various cell organelles [1]. Their expressions are regulated by many determinants including stress. Structural studies show that these folding catalysts mostly belong to either of three distinct families, namely, cyclophilins, FK506-binding proteins (FKBPs), and parvulins. Cyclosporin A, juglone, and FK506/rapamycin inhibit the enzymatic activities of cyclophilins, parvulins, and FKBPs, respectively [1]. The inhibitors cyclosporin A, FK506, and rapamycin are extensively used in the immunosuppressive therapy as these compounds along with their cognate PPIases block the activation of T-cells [1]. Conversely, juglone, a type of quinone, has long been employed to treat many microbial and inflammatory diseases [2].

PPIases belongs to three conserved families and are encoded by the most organisms including bacteria [1]. Escherichia coli, a representative Gram-negative bacterium, generates two cyclophilins (PpiA and PpiB), four FKBPs (FkpA, FkpB, FklB, and SlyD), and three parvulins (SurA, PpiC, and PpiD). Conversely, Bacillus subtilis, a model Gram-positive bacterium, produces two purvulins (PrsA and YacD), one cyclophilin (PpiB), and no FKBP. Trigger factor, a ribosome-associated chaperon with a FKBP domain, is, however, synthesized by both E. coli and B. subtilis. The E. coli-encoded SurA and PpiA appears to be the functional homologs of the B. subtilis-specific PrsA and PpiB, respectively. Bacterial PPIases usually exist in the periplasmic space, cytoplasmic membrane and in the cytoplasm [1]. These enzymes in bacteria are mostly involved in the maturation and transport of some secreted proteins. Several PPIases such as SurA, PrsA homolog PrsA2, trigger factor homolog RpoA, FkpA/FklB homolog Mip, PpiA and its homologs are associated with bacterial infections in human [1]. The existing inhibitors of PPIases are not useful for the treatment of bacterial infections, as they would block the enzymatic activities of the human counterparts. Additionally, cyclosporin A, FK506 and rapamycin would prevent the activation of T-cells. The high dose of juglone also appears to be very toxic to health [2]. Currently, several alternative strategies including the structural information of PPIases and their complexes are being harnessed to search for the non-toxic inhibitors capable of specifically blocking the bacterial PPIases [1].

Staphylococcus aureus, one of the leading bacterial pathogens, encodes a parvulin-type PPIase that shares significant identity with B. subtilis PrsA [3]. Deletion of the S. aureus PrsA encoding gene though did not affect its growth and shape dramatically enhanced the sensitivity of S. aureus to oxacillin and the glycopeptide antibiotics [3]. PrsA is directly regulated by the VraRS, a S. aureus-specific two-component system involved in the cell wall stress response [3]. Recently, this PPIase has been shown to be critical for maintaining the level of PBP2A, a peptidoglycansynthesizing enzyme with the reduced affinity to methicillin and other β-lactam antibiotics [3].

**Figure 1:**
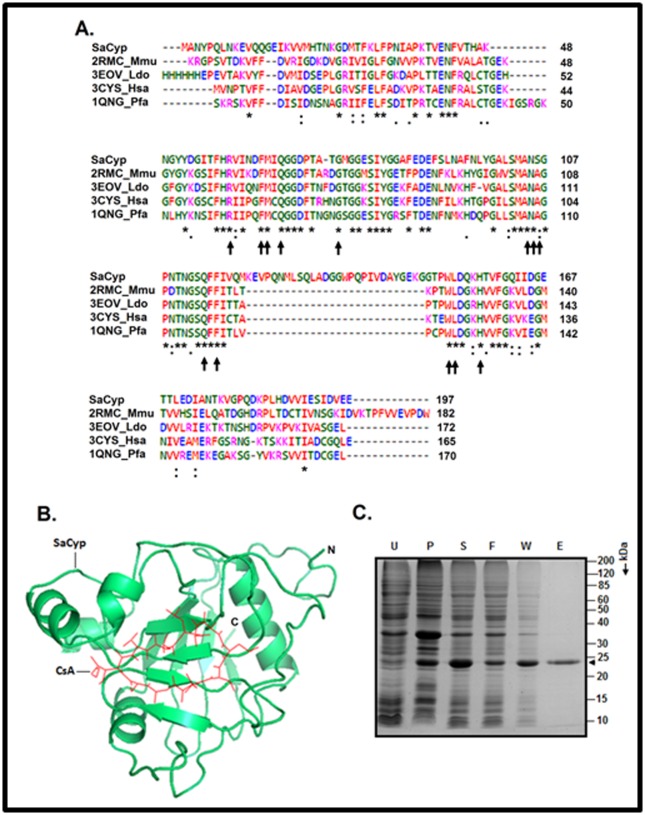
Identification and purification of S. aureus Cyp. (A) Alignment of the sequence of S. aureus-encoded Cyp (SaCyp) with those of some structurally known orthologous Cyps. The asterisk and colon indicate the conserved and the highly similar amino acid residues, respectively. The amino acid residues marked by arrows are involved in the binding of CsA. Abbreviations: 2RMC_Mmu, Murine cyclophilin C; 3EOV_Ldo, Leishmania donovani Cyp; 3CYS_Hsa, Homo sapiens Cyp, and 1QNG_Pfa, Plasmodium falciparum Cyp. All of the sequence/structural data reported here are available in NCBI/PDB database. (B) Three dimensional model structure of the SaCyp-CsA complex. The structure has been generated as described in Methodology. The ribbon, tube, and arrow denote α-helix, loop, and β-strand, respectively. N and C indicate N-terminal end and C-terminal end of SaCyp, respectively. (C) Purification of rCyp. Different protein containing fractions obtained from Ni-NTA chromatography are analyzed by SDS-13.5% PAGE. The uninduced, induced, supernatant, pellet, flow-thorough, wash, and elution fractions are loaded in lanes U, I, S, P, F, W, and E, respectively. Arrowhead indicates rCyp. Molecular masses of the marker (M) proteins (in kDa) are shown at the right side of the gel.

Our preliminary investigation suggests that the genome of S. aureus Newman [4] carries a gene for encoding a cyclophilin (Cyp)-like PPIase as well (Figure 1). The putative Cyp seems to be not essential for the in vitro growth of S. aureus [5] and is not induced by either heat or cold [6]. Thus far, no experiment was carried out to verify the enzymatic or the drug binding activity of likely Cyp from S. aureus Newman. In addition, little is known about the folding-unfolding mechanism or the stability of this tentative Cyp in the presence and absence of cognate inhibitor. Under the background of evolution and spread of drug-resistant strains of various microorganisms, the stability data of S. aureus Cyp may be useful for screening additional antimicrobial agents [7,8]. Herein, we have investigated a recombinant S. aureus Cyp (rCyP) using various spectroscopic probes. Our data reveal that rCyp not only specifically binds cyclosporin A (CsA) but also possesses PPIase activity. Binding of CsA to rCyp alters its structure, and shape to some extent. rCyp and rCyp-CsA in the presence of GdnCl appear to unfold via the formation of at least one intermediate. There is also substantial stabilization of rCyp in the presence of CsA.

## Methodology

### Basic molecular biological methods

Agarose gel electrophoresis, DNA and protein estimation, DNA sequencing, polymerase chain reaction (PCR), plasmid DNA purification, Western blotting, DNA cleavage with restriction enzymes, transformation, isolation of chromosomal DNA from S. aureus Newman, and SDS-PAGE have been carried out as demonstrated [9,10].

### Purification of rCyp

To purify the S. aureus Newman-encoded NWMN_0824 as a polyhistidine-tagged variant (designated as rCyp), the Newman genomic DNA was amplified using the oligonucleotides 824-1 (5’CTAGCTAGCGCTAACTATCCACAGTTAAAC) and 824-2 (5’ CCGCTCGAGTTATTCTTCAACATCAATAGATTC) as described [9]. The resulting 593 bp DNA fragment was cloned to plasmid pET28a (Novagen) using a standard method [9,10]. The yielded plasmid that carries no mutation in the cloned DNA insert was designated as p1350. Cloning has linked twenty-three extra amino acid residues (including six consecutive histidine residues) at the N-terminal end of SaCyp. Transforming E. coli BL21 (DE3) with p1350 as stated [9] created SAU1350.

Protein rCyp was purified from SAU1350 using a standard procedure with minor modifications [9]. Briefly, the IPTGinduced SAU1350 cells in buffer A [20 mM Tris-HCl (pH 8.0), 300 mM NaCl, 10 mM imidazole, 5% glycerol and 10 μg/ml PMSF] were ruptured followed by the purification of rCyp from the resulting supernatant by Ni-NTA affinity chromatography (Qiagen). The eluted rCyp was dialyzed against buffer B [20 mM Tris-HCl (pH 8.0), 1 mM EDTA, 300 mM NaCl, and 5% glycerol] as described [10]. The molar concentration of rCyp was determined using the molecular mass of its monomeric form.

### Structural investigation

To obtain clues about different structural properties of rCyp, we have performed intrinsic tryptophan (Trp) fluorescence spectroscopy, far-UV circular dichroism (CD) spectroscopy (200- 260 nm), and analytical gel filtration chromatography as reported earlier [9,10]. Different spectroscopic signals have also been collected for rCyp-CsA complex by the similar ways as stated above. The rCyp-CsA complex was made by incubating 10 μM rCyp with 20 μM cyclosporin A for 30 min at 4°C. The ratio of rCyp to cyclosporin A concentrations have been kept identical in all of the experiments reported here.

### Enzymatic activity and drug binding ability

The PPIase activity (kcat/Km) of rCyp in the presence or absence of different inhibitor (such as cyclosporin A, rapamycin, FK506, and juglone) was measured by RNase T1 (ribonuclease T1) refolding assay as stated [9,10]. The cyclosporin A binding affinity (Kd) of rCyp was determined by a procedure as followed to find out the rapamycin binding affinity of a recombinant FKBP22 [10].

### Unfolding of proteins

The rCyp and CsA-rCyp were treated with varying concentrations of GdnCl followed by the determination of the changes in their structural parameters both by intrinsic Trp fluorescence spectroscopy, and far-UV CD spectroscopy as described [10]. Refolding of GdnCl-denatured proteins was monitored by intrinsic Trp fluorescence spectroscopy.

### Thermodynamic parameters of unfolding

Presuming that the GdnCl-induced protein unfolding follows a two-state model (N ↔ U) [11], the associated parameters such as fu, the fraction of unfolded protein molecules, Cm, GdnCl concentration at the midpoint of denaturation (i.e. concentration of GdnCl when ΔG = 0) , ΔGW, free energy at 0 M GdnCl, m, cooperative parameter of unfolding, and ΔΔG, the difference of free energy alteration between rCyp and rCyp-CsA, were measured as stated [11].

### Statistical and computational studies

Sequences of Bacillus subtilis-encoded PpiB [1] and the related cyclophilins were downloaded from NCBI, USA. SignalP 4.1, ClustalW, and blastP program carried out signal sequence prediction, sequence alignment, and sequence similarity search, respectively.

The tertiary model structure of SaCyp, developed using ITASSER server, was analyzed by Amber 10 [12] to minimize its energy. The structure of cyclosporin A was extracted from the structure of a murine cyclophilin-cyclosporin A complex (PDB ID: 2RMC) [13]. Both the structures of SaCyp and cyclosporin A were converted to the respective PDBQT format using AutoDockTools. Finally, the processed structures were docked using AutoDock Vina. Using AutoDock vina with the energy range of 4 kcal/mol generated a total of five ligand-docked protein structures. The docked structure with the maximum number of hydrogen bonds, and interactions was selected for study. A three-dimensional box with the size of 25 Å X 25 Å X 25 Å in the complex carries all of the interacting residues.

The data reported here are the means of at least three different studies with standard deviation. The p values, standard deviation, and mean were determined as stated [10]. Two results are significant if the related p value is <0.05.

## Result and discussion

### Identification of a putative cyclophilin from S. aureus

To identify whether a cyclophilin-like PPIase is encoded by S. aureus, we have carried out a blastP analysis using the sequence of a B. subtilis PpiB, one of the first discovered cyclophilins from a Gram-positive bacterium [1]. To minimize the number of PpiB orthologs in S. aureus, we have searched only the genome of S. aureus Newman, a virulent S. aureus strain that shows sensitivity to methicillin [4]. The analysis yielded a S. aureus protein (namely, NWMN_0824) that showed 39% identity with the B. subtilis PpiB at the amino acid sequence level. An identical or nearly an identical homolog of NWMN_0824 has been noticed in all other sequenced S. aurues strains (data not shown). The amino acid sequence of NWMN_0824 (designated SaCyp) also exhibited significant identity with those of cyclophilins encoded by various living organisms including human (data not shown).

The putative S. aureus SaCyp apparently carry no signal peptide sequence or a transmembrane domain, indicating that it could be a cytoplasmic protein. However, SaCyp possesses a putative cyclosporin A (CsA) binding domain that is composed of amino acid residues 17-195. To gain additional clues about the tentative CsA binding domain, we have aligned the sequence of SaCyP with those of some selected homologs having known structures (Figure 1A). Of the thirteen conserved CsA binding amino acid residues [1], twelve residues are also observed in SaCyp. The only discrepancy resulted in due to the alignment of Ser106 in SaCyp with a CsA binding Ala residue in the homologs (Figure 1A). However, the Ser106 also shows alignment with Gln, Asn, and Cys residues belonging to other cyclophilin homologs (data not shown) [1].

Cyclophilins usually possess a β-barrel conformation that is formed by eight anti-parallel β-strands and a flanking α-helix at each side [1]. A hydrophobic active site made by most of the β- strands and connecting loops is located in one face of such PPIase. The majority of the amino acid residues involved in the binding of CsA are also critical for the formation of the active site in the cyclophilins [1]. To see whether SaCyp adopts a similar conformation, a three-dimensional model structure of this protein was developed as described in Methodology. A model structure of CsA-bound SaCyp has been produced as well (Figure 1B). The results indicate that SaCyp, like other cyclophilins [13], also contains the basic β-barrel structure and the CsA binding site. Thus, the computational analyses together suggest that SaCyp could be a potential CsA binding PPIase.

### Purification of a recombinant SaCyp

To determine the structure, function and stability of SaCyp, a polyhistidine-tagged variant of this protein (designated rCyP) was purified by an affinity chromatography as described in Methodology. Different fractions accrued from the chromatography were analyzed by a 13.5% SDS-PAGE (Figure 1C). The elution fraction largely contains a single protein band having the molecular mass of ~24 kDa. The theoretical molecular mass of rCyp has been computationally determined to be 24.07 kDa. Additional Western blot analysis reveals the interaction between the ~24 kDa protein and the anti-his antibody (data not shown). Taken together, the data suggest that the ~24 kDa protein in the eluted fraction may be rCyp.

### Biological activity of rCyp

**Figure 2:**
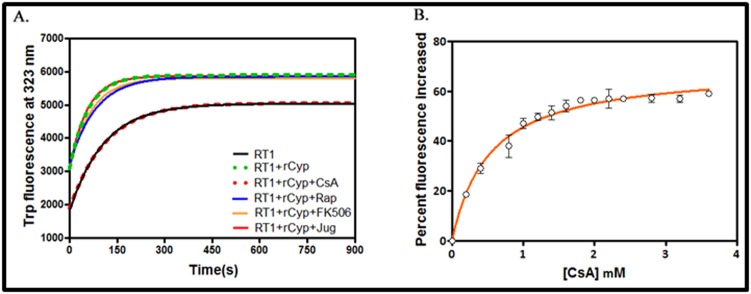
Function of rCyp. (A) RNase T1 refolding assay. The curves represent the change in Trp fluorescence intensity of denatured RNase T1 (RT1) with time in the absence and the presence of rCyp. The rCyp-mediated refolding of RT1 has also been studied separately in the presence of 10 folds molar excess of each of cyclosporin A (CsA), rapamycin (Rap), FK506 and juglone (Jug). (B) Cyclosporin A binding assay. The curves indicate the increase of Trp fluorescence intensity of rCyp in the presence of 0-3.6 μM of CsA. rCyp concentration used here is 2 μM.

To determine whether the putative SaCyp possesses any PPIase activity, we have performed an RNase T1 refolding assay using rCyP by a standard method [9]. Figure 2A shows the comparatively rapid refolding of the denatured RNase T1 in the presence of rCyp. The catalytic activity (kcat/Km) value of rCyp determined from Figure 2A is about 5 ± 0.05 μM-1min-1. To prove that rCyp is truly a cyclophilin, we separately checked the PPIase activity of rCyp in the presence of CsA, juglone, rapamycin, and FK-506. As shown in Figure 2A, the PPIase activity of rCyp was only inhibited by CsA, suggesting that it is surely a cyclophilin.

To determine the inhibitor binding affinity (Kd) of rCyp, we have measured the Trp fluorescence change of this PPIase in the presence of varying concentrations of CsA (Figure 2B). The resulting Kd value for the rCyp and CsA interaction is about 0.53±0.05 μM, indicating that this protein binds CsA with an appreciable affinity.

### Effects of CsA on the structure, shape and size of rCyp

**Figure 3:**
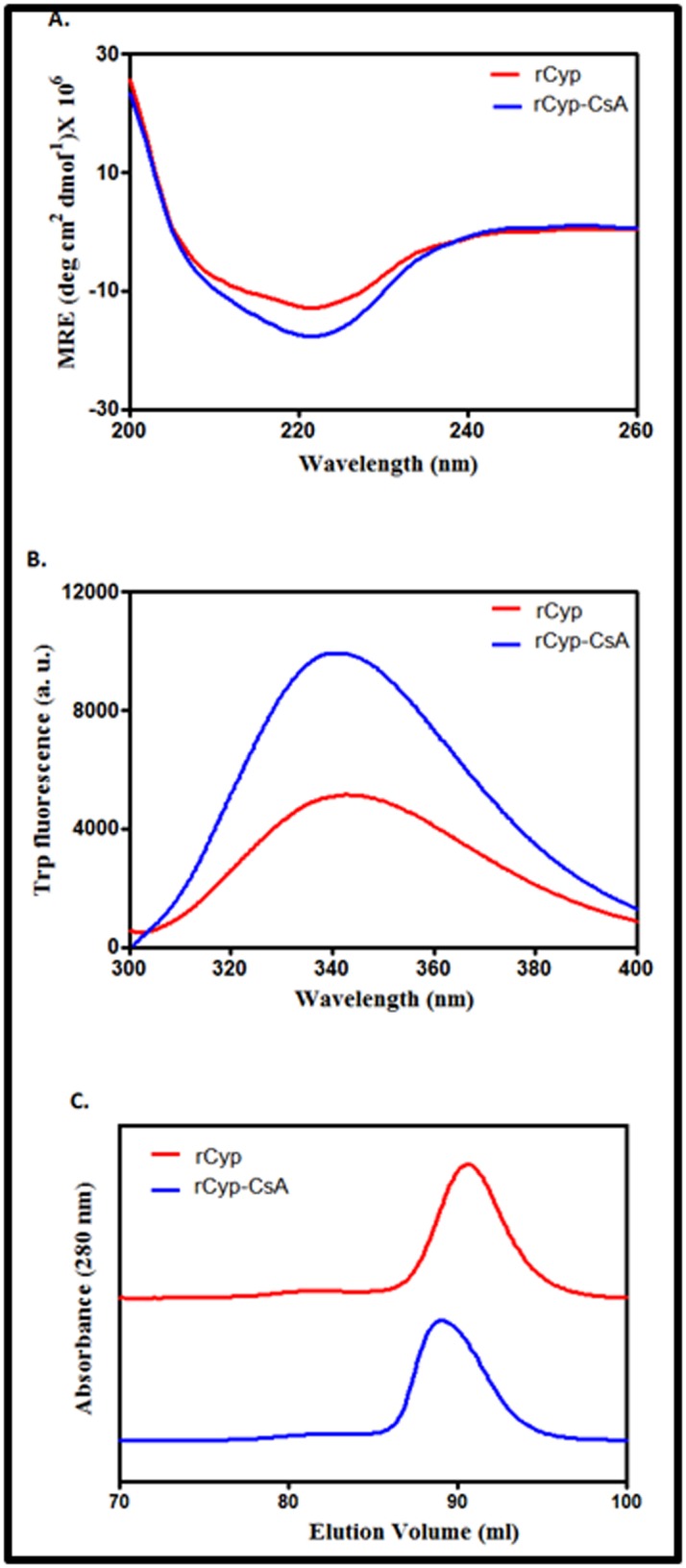
Structure and shape of proteins. Far-UV CD spectra (A), intrinsic Trp fluorescence spectra (B), and gel filtration elution profiles (C) of rCyp and CsA-bound rCyp.

Binding of CsA to cyclophilins usually alters their structure [1]. To determine whether the binding of rCyp also changes its structure, we recorded the far-UV CD and Trp fluorescence spectra of this cyclophilin both in the presence and absence of CsA. Figure 3A shows that the far-UV CD spectrum (200-260 nm) of rCyp differs from that of CsA-equilibrated rCyp (rCyp-CsA). However, both spectra carry a peak at ~222 nm, indicating the presence of α helix in these proteins. A computational analysis indicates that rCyp and rCyp-CsA are composed of ~19% and ~25% α-helix, respectively. The CsA-bound and unbound rCyp also carry variable extent of β-strand (data not shown). Taken together, binding of CsA to rCyp alters its secondary structure to some extent.

To obtain clues about the effects of CsA on the tertiary structure of rCyp, the intrinsic Trp fluorescence spectra of rCyp and rCyp- CsA were recorded after excitation at 295 nm. Figure 2B shows that the emission maximum (λmax) of rCyp and rCyp-CsA are ~343 and ~341 nm, respectively. In addition, the fluorescence intensity of rCyp has been increased about two folds in the presence of CsA. In sum, interaction between CsA and rCyp has altered the structure of the latter.

To check the oligomeric status of rCyp and rCyp-CsA in solution, we have performed an analytical gel filtration chromatography as described in Methodology. Figure 2C shows that passage of CsAbound and CsA-unbound proteins through the gel filtration column have yielded a single peak with the distinct elution volume. While the elution volume of rCyp corresponds to 90.5 ml, that of rCyp-CsA is 89.07 ml. Comparing these elution volumes with those of various monomeric proteins (data not shown), the apparent molecular masses of rCyp and rCyp-CsA have been determined to be 23.77 and 26.9 kDa, respectively. The molecular mass of rCyp, calculated from its amino acid sequence, is about 24 kDa. The results together indicate that both CsAbound and CsA-unbound rCyp primarily exist as the monomers in solution. The molecular mass of CsA is about 1.2 kDa. The data therefore suggest that the binding of CsA has slightly enhanced the shape of rCyp.

### Unfolding of CsA-bound/unbound rCyp

**Figure 4:**
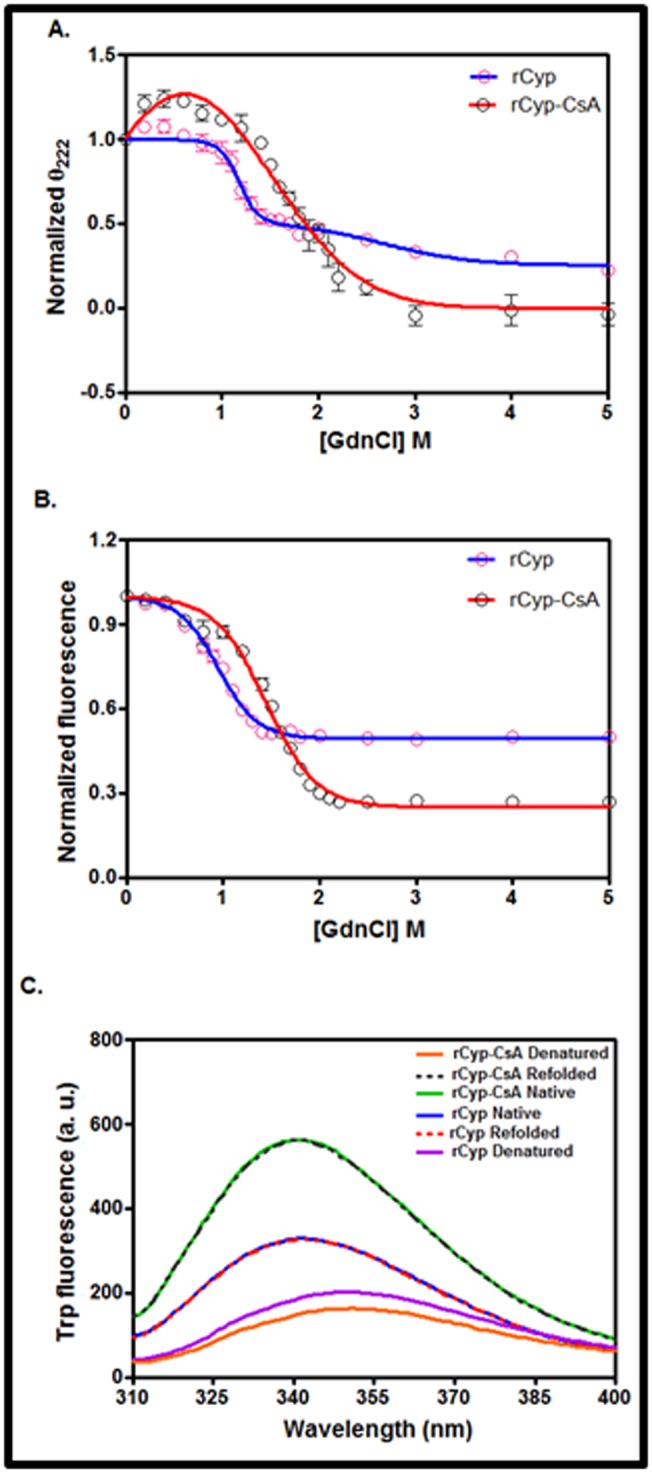
Unfolding of proteins. (A) The curves show the change in ellipticity value of rCyp and CsA-bound rCyp at 222 nm (θ222) in the presence of 0-5 M GdnCl. All of the values, extracted from the respective spectra of rCyp and CsA-equilibrated rCyp (data not shown), are normalized as reported earlier [9, 10]. (B) The curves show the alteration of Trp fluorescence intensity values (at 342 nm) of rCyp and CsA-bound rCyP at 0-5 M GdnCl. All curves are best-fit curve. (C) Refolding of GdnCl-denatured proteins.

Some cyclophilins in the presence and absence of CsA were unfolded via the synthesis of at least one intermediate [14,15]. To understand whether the unfolding of SaCyp in the presence and absence of cognate drug occurs by a similar mechanism, we have separately investigated the GdnCl-induced unfolding of rCyp and rCyp-CsA by the far-UV CD and intrinsic Trp fluorescence spectroscopy. The resulting denaturation curves, produced using the ellipticity values of rCyp and rCyp-CsA at 222 nm, are presented in Figure 4A. rCyp shows a biphasic curve, whereas, rCyp-CsA yields neither a biphasic nor a monophasic curve at 0-5 M GdnCl. Conversely, the curves, formed using the Trp fluorescence intensities of rCyp and rCyp-CsA, are monophasic in nature under identical conditions (Figure 4B). The associated λmax values of rCyp and rCyp-CsA have gradually started increasing with the concomitant decrease of their fluorescence intensity values (data not shown). Finally, their λmax values have reached to 350 nm when there was no further reduction of fluorescence intensity. The Trp fluorescence spectroscopy also clearly shows that the initiation and termination of unfolding of rCyp-CsA have occurred at relatively higher GdnCl concentrations.

To check the reversibility of the GdnCl-induced denaturation of rCyp or rCyp-CsA, we have recorded the Trp fluorescence spectra of the denatured, native, and the likely refolded forms of these proteins as described in Methodology. The resulting data reveal nearly a complete overlapping of the Trp fluorescence spectrum of the refolded proteins with those of their native counterparts (Figure 4C). Together, the GdnCl-induced unfolding of rCyp and rCyp-CsA are completely reversible in nature.

### Mechanism of unfolding of the drug-bound/unbound rCyp

The dissimilar pattern of unfolding curves originated from two different spectroscopic probes (Figure 4) indicates that there may be generation of some rCyp/rCyp-CsA intermediate(s) in the presence of GdnCl. Two lines of evidences indeed have supported the above proposition. We noticed that the curves produced by plotting the fraction of denatured rCyp/rCyp-CsA (estimated from Figure 4) against the corresponding GdnCl concentrations did not overlap with each other (data not shown). Previously, the non-coincidence of such denaturation curves was used to predict the synthesis of unfolding intermediates of many proteins [16]. Secondly, the phase diagrams [10], an indicator of the hidden intermediates of proteins, have been developed by plotting the Trp fluorescence intensities of rCyp/rCyp-CsA at 320 nm against their Trp fluorescence intensities at 365 nm (data not shown). The non-linear plots obtained for both rCyp and rCyp- CsA indicate that these macromolecules have generated some intermediate(s) in the presence of GdnCl.

To obtain clues about the number of intermediates formed by rCyp and rCyp-CsA, the related unfolding curves derived from the ellipticity data are analyzed using different models as well [11,16]. The CD values of rCyp, unlike the fluorescence values of this protein, fit best with the three-state equation that yielded the Cm values of 1.18±0.02 and 2.67±0.35 M. Such data are indicative of the formation of one rCyp intermediate most likely at ~1.5 M GdnCl. Conversely, rCyp-CsA may generate multiple intermediates as the matching CD data did not show fitting with either the two-state or the three-state equation. The ellipticity value of rCyp-CsA is increased about 23% upon raising the GdnCl concentrations from ~0 to 0.6 M. Subsequently, the ellipticity values are reduced nearly 90% upon further raising the GdnCl concentrations to ~3 M, indicating that the unfolding of rCyp-CsA primarily occurs at ~0.6 - 3 M GdnCl. The λmax value of rCyp-CsA is increased about 1 nm at 0.6 M GdnCl, suggesting that rCyp-CsA possesses a slightly altered tertiary structure at this GdnCl concentration. Taken together, one of the rCyp-CsA intermediates may be formed at ~0.6 M GdnCl. The λmax value of rCyp intermediate appears to be 347 nm, indicating that this intermediate, unlike the rCyp-CsA intermediate, remains mostly as an unfolded form.

### CsA-induced stabilization of rCyP

Binding of a ligand to the cognate protein usually stabilizes it [17]. To verify whether the binding of CsA stabilizes rCyp, we have analyzed the monophasic unfolding curves using a twostate equation [11]. Table 1 shows the values of resulting thermodynamic parameters, namely, Cm, m, ΔGW, and ΔΔG. Additional analyses reveal that the Cm value of rCyp-CsA is considerably greater than that of rCyp (p = 0.009). The free energy change ΔΔG between rCyp-CsA and rCyp is about 1.22±0.16 kcal mol-1 (Table 1). The data together suggest the considerable thermodynamic stabilization of rCyp in the presence of CsA. As suggested for other proteins [7,8], the CsA-mediated stabilization of rCyp may guide to screen the new antimicrobial agents in the future.

## Conclusion

The genome of S. aureus Newman harbors a putative cyclophilin (SaCyP)-encoding gene. The present investigation has revealed that rCyp, a recombinant SaCyp, possesses a PPIase activity, which is specifically inhibited by cyclosporin A (CsA). Both CsAbound rCyp and rCyp exist as the monomers in the aqueous solution. Binding of CsA to rCyp has, however, changed its structure, and shape to more than 10%. The GdnCl-induced equilibrium unfolding of CsA-bound rCyp or rCyp occurs via the formation of at least one intermediate. In addition, binding of CsA to rCyp has stabilized this protein substantially. The CsAinduced stabilization of rCyp may be useful for screening the new antimicrobial agents in the future.

## Note added in proof

The authors while preparing the manuscript have noticed that the cloning of a cyclophilin from Staphylococcus aureus USA300 has been reported by a research group [ J Bacteriol. 2016 Dec 13; 199(1): e00453-16].

## Conflict of Interest

The authors declare no conflict of interest.

## Figures and Tables

**Table 1:** Different thermodynamic parameters^a^

Protein/ protein-drug	Cm (M)	m (kcal mol-1M-1)	ΔGW (kcal mol-1)	ΔΔG (kcal mol-1)
rCyp	0.94±0.01	2.83±0.21	2.65±0.17	
rCyp-CsA	1.41±0.01	2.32±0.25	3.28±0.38	1.22±0.16
